# RHOA and mDia1 Promotes Apoptosis of Breast Cancer Cells Via a High Dose of Doxorubicin Treatment

**DOI:** 10.1515/biol-2019-0070

**Published:** 2019-12-31

**Authors:** Peter Bober, Michal Alexovič, Zuzana Tomková, Róbert Kilík, Ján Sabo

**Affiliations:** 1 Department of Medical and Clinical Biophysics, Faculty of Medicine, University of P.J. Šafárik in Košice, Trieda SNP1, 04011 Košice, Slovakia; 2 1st Department of Surgery, Faculty of Medicine, University of P.J. Šafárik in Košice, Trieda SNP1, 04011 Košice, Slovakia

**Keywords:** Transforming RhoA protein, Diaphanous homolog 1 protein, actin cytoskeleton, stress fibre

## Abstract

**Background:**

Transforming RhoA proteins (RHOA) and their downstream Diaphanous homolog 1 proteins (DIAPH1) or mDia1 participate in the regulation of actin cytoskeleton which plays critical role in cells, i.e., morphologic changes and apoptosis.

**Methodology:**

To determine the cell viability the real time cell analysis (RTCA) and flow cytometry were used. To perform proteomic analysis, the label-free quantitative method and post-translation modification by the nano-HPLC and ESI-MS ion trap mass analyser were used.

**Results:**

The results of the cell viability showed an increase of dead cells (around 30 %) in MCF-7/DOX-1 (i.e., 1μM of doxorubicin was added to MCF-7/WT breast cancer cell line) compared to MCF-7/WT (control) after 24 h doxorubicin (DOX) treatment. The signalling pathway of the Regulation of actin cytoskeleton (p<0.0026) was determined, where RHOA and mDia1 proteins were up-regulated. Also, post-translational modification analysis of these proteins in MCF-7/DOX-1 cells revealed dysregulation of the actin cytoskeleton, specifically the collapse of actin stress fibbers due to phosphorylation of RHOA at serine 188 and mDia1 at serine 22, resulting in their deactivation and cell apoptosis.

**Conclusion:**

These results pointed to an assumed role of DOX to dysregulation of actin cytoskeleton and cell death.

## 1 Introduction

Doxorubicin (DOX) is one of the most useful anticancer agents. However, the use of higher DOX doses is limited by the cardiotoxicity [1, 2, 3]. DOX may intercalate into DNA and inhibits the progression of topoisomerase II, inducing its cytotoxicity [4]. Under cytotoxic stress conditions, Rho GTPase signalling cascade results in actin cytoskeleton remodelling [5]. Rho family GTPases are therefore key regulators of the actin cytoskeletal dynamics [6]. It was also proved that DOX induces actin cytoskeleton alterations, namely through Rho family RhoGTPase pathways [7].

The structural mechanisms of RHOA, RAC1 and CDC42 proteins are well documented, as are their regulatory partners and post-translational modifications [8]. Moreover, it has been known that RHOA is phosphorylated via multiple kinases including cAMP-dependent protein kinase (PKA) and cGMP-dependent protein kinase (PKG) [9, 10]. These kinases phosphorylate RHOA at serine-188 and deactivate RHOA by increasing its interaction with Rho GDP-dissociation inhibitor (RhoGDI), i.e. translocation of RHOA from membrane to cytosol [11] independent of GDP±GTP cycling [12].

mDia1 is a member of the diaphanous-related formin family of Rho effector proteins [13]. Unlike the deactivation mechanism of RhoA, the auto-inhibition of mDia1 through its phosphorylation at serine-22 has still not been fully elucidated.

RHOA and mDia1 deactivated and auto-inhibited proteins in this order collapse the actin stress fibbers [11, 14]. The actin stress fibber disassembly is associated with the effect of high dose of DOX, resulting in cellular apoptosis [15]. Our results confirmed that the high dose of DOX (1μM) during a 24 h treatment had an impact on the cell viability and proteins deactivation via phosphorylation of mDia1 and RHOA on Ser-188 and Ser-22 in this order.

## 2 Materials and methods

### 2.1 Cell culture

Biological triplicates of MCF-7/WT and MCF-7/DOX-1 (ATCC HTB-22, USA) were cultured during 24 h. in a 75 cm^2^ culture flask (Becton, Dickinson and Company, USA). The 10 mL of culture medium consisted of Dulbecco’s Modified Eagle Medium (DMEM, 4.5 g L^-1^ Glucose, Lonza-BioWhittaker, Belgium) and F-12 HAM medium (Sigma, USA), prepared in a 1:1 (v/v) ratio. This mixture was supplemented using 5% FBS (GIBCO, USA) and 1% Sodium Pyruvate 100 mM (GIBCO, USA).

### 2.2 xCELLigence cell proliferation assay

In the first step, the 100 μL of culture medium was pipetted into a 16-well E-plate. The plate containing medium was inserted in the measuring device, i.e. xCELLigence system (ACEA Biosciences, USA). Subsequently, 2x10^4^ MCF-7 cells were pipetted into the wells. Once the cells reached one half log phase (i.e., after 21 h and 35 min), DOX was added in the range 0.001-1 μM up to a total volume of 250 μL. Using the Real Time Cell Analysis (RTCA) software the half maximal inhibitory concentration (IC_50_) values were determined during a 24 h treatment.

### 2.3 Flow cytometry

The viability of MCF-7/WT and MCF-7/DOX-1 cells (biological duplicates) at a density of 8x10^4^ cells/well were measured via the Muse Cell Analyzer (Millipore, Hayward, CA, USA) using the Muse™ Count and Viability Kit (Millipore, Hayward, CA, USA) after 24 h. Total volume of the cells was 1 mL.

### 2.4 Proteins extraction and in-solution digestion

MCF-7/WT and MCF-7/DOX-1 cells (biological triplicates) were lysed by an 8 M urea (100 mMTris/HCl, pH 8) solution. Lysed cells were centrifuged for 15 min at 12000g and 4°C. Glacial acetone (-20°C) in a ratio of 1:5 (v/v) was added to the supernatant. Afterwards the solution was vortexed, stored at -20°C (60 min) and centrifuged. The protein pellets were ultimately dried using a Vacuum concentrator (Labconco, USA) and dissolved in 8 M urea (100 mMTris/HCl, pH 8). The Bradford method and UV-Vis 3600 spectrophotometer (Shimadzu, Japan) determined protein concentration. Then a 1.1 % of 0.1 M DTT (100 mMTris/HCl, pH 8) solution was added to the dissolved proteins and incubated at the light at 37°C for 30 min. Subsequently, the 0.1 % of 0.5 M IAA (100 mMTris/HCl, pH 8) solution was added and incubated in the dark at 37°C for 30 min. Then, the glacial acetone (-20°C) was added and left for 60 min in a freezer at -20°C and centrifuged at 4000g for 50 min. The pellet was subsequently dried and dissolved in 8 M urea (100 mMTris/HCl, pH=8). After that, 2 mM of calcium chloride (10 mMTris/HCl, pH=8) and trypsin in ratio 1:100 (w/w) ratio were added and incubated at 37°C for 12 h. trifluoroacetic acid (TFA) 20% was finally used to stop digestion of proteins. The final concentration of proteins in solution was 1 μg μL^-1^. Peptides were ultimately desalted by solid phase extraction using C_18_ matrix spin cartridges (Agilent Technologies, USA).

### 2.5 Off-gel fractionation

The 1 mg of solution containing peptides was inserted to an Agilent 3100 OFFGEL Fractionator (Agilent Technologies, USA) for electromigration-based fractionation. Peptides were resuspended in a 5% glycerol and a 1% ampholyte (GE Healthcare Life Sciences, USA), applied to immobilized pH gradient (3–10) by using 12 cm strips (GE Healthcare Life Sciences, USA) and focused at default conditions with 24 hour runtimes.

### 2.6 LC-MS/MS analysis

MCF-7/WT and MCF-7/DOX-1 samples were subjected to a nano-liquid HPLC system (Ultimate 3000 RSLC Nano, Thermo Fisher Scientific, USA) on-line coupled with an amaZon speed ETD ion trap mass spectrometer (Bruker Daltonik, Germany). The CaptiveSpray (Bruker Daltonik, Germany) was used as the ion source. The procedure was started with a 1 μL sample injection. Consequently, peptides (the 1 μg solution) were loaded to an Acclaim® PepMap 100 C_18_ trap column (5 μm particles, 100 Å, 300 μmi.d. × 2 cm) via a mobile phase (MF, 98% water, 2% ACN containing 0.1% formic acid - FA) at 8 μL min^-1^ flow-rate. Then the peptides were washed out from a trapping column to an Acclaim® PepMap RSLC analytical column C_18_ (2 μm particles, 100 Å, 75 μmi.d. × 15 cm) and mass spectrometer by MF A (98% water, 2% ACN and 0.1% FA) and MF B (95% ACN, 5% water containing 0.1% FA). The gradient elution starting from 96%:4% to 65%:35% (MFA: MFB) was reached in a 100 min run at 0.4 μL min^-1^ flow-rate. For MS spectra, the acquisition parameters were: positive ionization mode, enhanced resolution mode, Ion charge control (ICC). The target was set up to 400 000 compounds, maximum accumulation time was set up to 50 ms and scan range was 300-1300 m/z. For MS/MS spectra, acquisition parameters were used: Xtreme resolution mode, ICC target was put up to 500 000 compounds, maximum accumulation time was put up to 100 ms and isolation width was 2.2 m/z. Once a precursor ion was selected for one MS/MS spectrum, its active exclusion was performed within a 0.25 min release time.

### 2.7 Protein databases search

The acquired raw data were assessed via a Compass Data Analysis software (version 4.2, Bruker Daltonik GmbH, Germany). ProteinScape software (version 3.1.2 450, Bruker Daltonik GmbH, Gemany) served for searching proteins against the SwissProt database utilising the Mascot search engine (version 2.4.0, Matrix Science, London, UK), under the following conditions: taxonomy – Homo sapiens (human); enzyme – Trypsin, fixed modifications – Carbamidomethyl (C) and variable modifications – Oxidation (M), Phosphorylation (R), Phosphorylation (Y) and Phosphorylation (ST); allowed missed cleavages - up to 2, peptide charge +2, +3; minimum peptide length – 3, protein assessment: False discovery rate (FDR) was < 1%, with minimally 2 unique peptides.

### 2.8 Protein identification, label-free quantification and pathway analysis

The Scaffold 4.7.5 (Trial Version, Proteome Software Inc., Portland, OR, USA) software was used to validate MS/MS based peptide and protein identifications. To perform the label-free protein quantification data were normalised and a quantitative method was used, i.e., the Total Spectra Count. The significantly impacted pathways in the MCF-7/DOX-1 treatment compared to MCF-7/WT cells were analysed using Advaita Bioinformatic iPathwayGuide software in the context of pathways obtained from the Kyoto Encyclopedia of Genes and Genomes (KEGG) database.

### 2.9 Post-translation modification and molecular modelling

For the in depth analysis of phosphosites located in MS/MS spectra the Scaffold PTM (Trial Version 3.0.0, Proteome Software Inc.) was used. The PyTMs designed to facilitate the introduction of post-translational modifications into existing 3D-structure models was implemented as a plugin for the PyMOL Molecular Graphics System (Version 2.0, Schrödinger, LLC, http://www.pymol.org/)

### 2.10 Statistical analysis

The statistical data of biological duplicates by flow cytometry were represented as the corresponding standard deviations (Student’s t-test, α < 0.05). Peptide identifications were accepted if they could be established at greater than 95.0% probability by the Peptide Prophet algorithm using Scaffold delta-mass correction. Protein identifications were accepted if they could be established at greater than 99.0% probability and contained at least 2 identified peptides. Protein probabilities were assigned by the Protein Prophet algorithm. To the label-free quantification and pathways analysis of biological triplicates was applied the Fisher’s Exact Test (α < 0.05) from two experimental groups - MCF-7/WT (control group) and MCF-7/DOX-1 (treatment group). The phosphosite localisation probabilities were calculated using the Ascore probability-based scoring technique by accepting only the sites that were significant at α < 0.05.

## 3 Results

### 3.1 The cytotoxicity of DOX in MCF-7 cells

**Figure 1:**
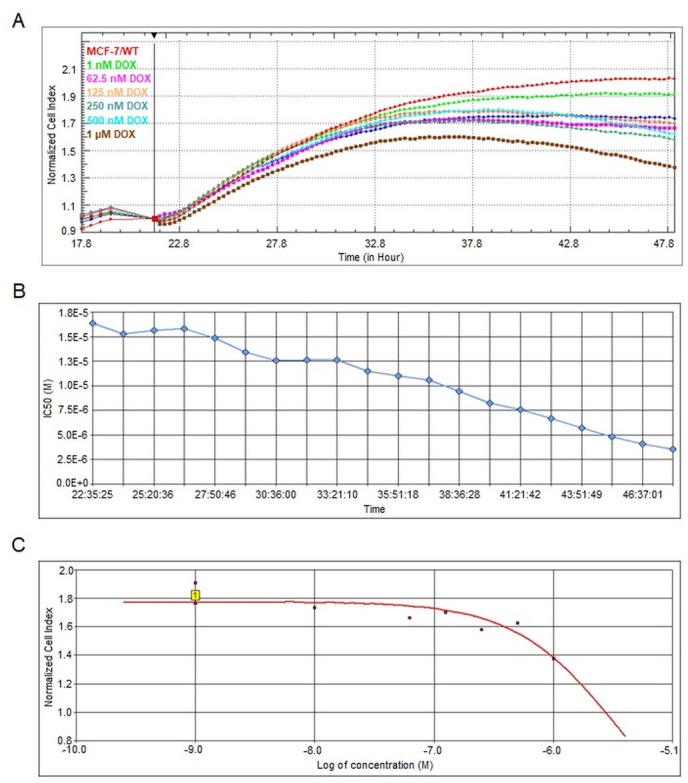
The real-time monitoring of DOX cytotoxic effect in MCF-7 cells. (A) Normalized cell index values of MCF-7 cell line after addition of DOX in the range of 0,001-1 μM. (B) The time dependent IC_50_ of DOX (time range: 21:35:30 ~ 48:07:13) (C) The Dose-Response Curve (CI at a time point vs conc.) of the highest cytotoxicity of DOX (IC_50_ = 3.53) after 24 h treatment.

The cell index (CI) of the MCF-7 cell line was monitored and normalized (normalized time; 21 h and 35 min) after the addition of different DOX concentrations in the range of 0.001-1 μM. As shown in Fig. 1A, the decreased CI was observed with the increase of DOX concentration. The highest decrease in CI value and thus cell proliferation was measured with maximal DOX concentration, i.e., 1μM after 12 h (see brown line). Also, as can be seen in Fig. 1B, the half maximal inhibitory concentration (IC_50_) of DOX in MCF-7 cells gradually decreased with time. The highest toxicity of DOX was observed after a 24 h treatment (time: 48 h and 07 min, IC_50_=3.53 μM) as indicated in Fig. 1B and 1C. Therefore it has been confirmed that concentration and exposure time of the DOX are the main factors related to proliferation of MCF-7 cells.

### 3.2 Cell viability of MCF-7 cells

**Figure 2:**
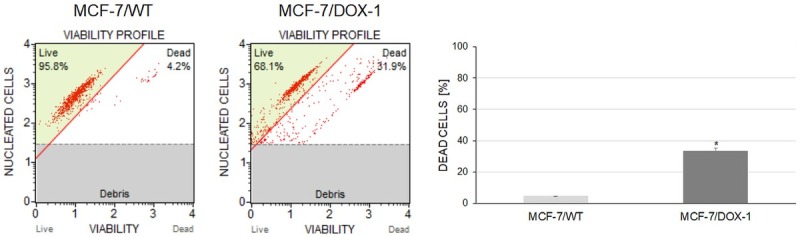
Determination of the number of MCF-7 cells and cell viability using Muse™ Cell Analyzer. Each sample was subjected to run in duplicate. Error bars represent standard deviation. Significant differences relative to the control are marked with an * (*p* < 0.05).

To confirm the cytotoxic effect of DOX on MCF-7 cells at 1 μM, the cell viability was measured by flow cytometry approach. The viability of the MCF-7/WT and MCF-7/DOX-1 cells were observed using the Muse™ Count and Viability Kit after 24 hours treatment. From the results, it was observed that the count of MCF-7/DOX-1 dead cells was increased at about 30% after 24 h DOX treatment compared to MCF-7/WT cells as depicted in Fig. 2.

### 3.3 Protein quantification and validation

1 μM concentration of the DOX was used on the MCF-7 cells based on the highest cytotoxic effect (Fig. 1A). Using Scaffold software 1772 proteins were identified with different regulations from two experimental groups, i.e., MCF-7/WT (control group) and MCF-7/DOX-1 (treatment group), each performed three times. The label-free quantitative proteomic analysis utilising the Normalised Total Spectral Count quantification method showed statistically significant changes in 366 proteins, i.e., those passing the T-test (p ≤ 0.05). Among them, the proteins with positive regulation of apoptotic process; Cell cycle and apoptosis regulator protein 2 (CCAR2), Apoptosis-inducing factor 1, mitochondrial (AIFM1), Programmed cell death 6-interacting protein (PDCD6IP) were up-regulated (Tab.1)

**Table 1:** Up-regulation of RHOA, mDia1 and proteins with positive regulation of apoptotic process (PDCD6IP, AIFM1, CCAR2) in MCF-7/DOX-1 cells utilising the Normalised Total Spectra quantification method (T-test, p≤0.05).

Accession	Gene	Protein	MCF-7/WT			MCF-7/DOX-1			T Test	MCF-7/WT vs MCF-7/DOX-1
			
			I. (NTS)*	II. (NTS)	III. (NTS)	I. (NTS)	II. (NTS)	III. (NTS)	p≤0.05	log_2_FC
Transforming protein RhoARHOA		RHOA_HUMAN	0	0	0.99	5.26	3.43	8.53	0.02	4.1
Protein diaphanous homolog 1	DIAPH1	DIAP1_HUMAN	0	0	0.99	1.50	6.00	7.58	0.05	3.9
Programmed cell death 6-interacting protein	PDCD6IP	PDC6I_HUMAN	6.09	1.05	2.99	26.31	18.02	15.16	0.01	2.6
Apoptosis-inducing factor 1, mitochondrial	AIFM1	AIFM1_HUMAN	2.03	2.09	1.99	11.28	9.44	5.68	0.02	2.1
Cell cycle and apoptosis regulator protein 2	CCAR2	CCAR2_HUMAN	0	0	0.99	9.02	6.87	16.10	0.02	5.0

*NTS: Normalised Total Spectra method uses the sum of all the spectra associated with a specific protein within a sample which includes also those spectra that are shared with other proteins

### 3.4 Signalling pathways analysis

**Figure 3:**
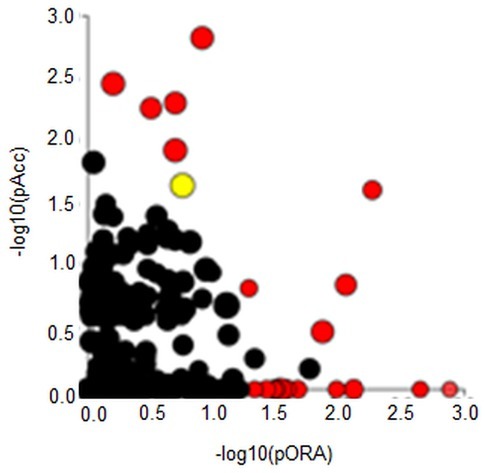
Signalling pathways analysis on the all 1772 identified proteins (log2-fold change >0.6, p ≤ 0.05) via iPathwayGuide analysis tool.

**Figure 4:**
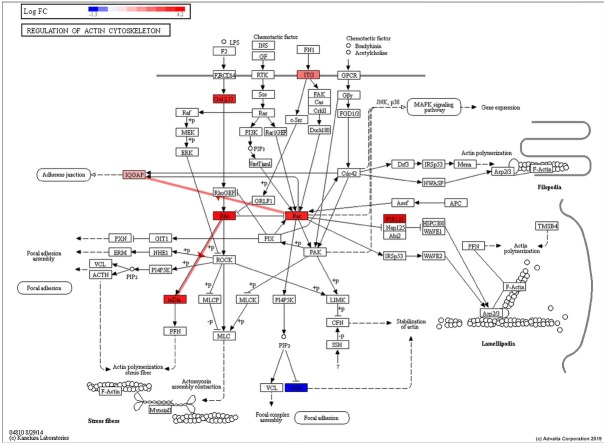
The regulation of actin cytoskeleton obtained from the KEGG pathways database (p < 0.0026). The 8 proteins like: RAC1, CYFP1, GNA13, IQGAP1, ITGB1, RHOA, GSN and DIAPH1 with different regulation were identified.

Signalling pathways analysis was done on the all 1772 identified proteins (log_2_-fold change >0.6, p ≤ 0.05) using iPathwayGuide analysis tool that uses two types of evidence, i.e.: over-representation on the horizontal axis (pORA) and perturbation on the vertical axis (pAcc). As shown in Fig. 3, 21 significant pathways (p ≤ 0.05) are shown in red, whereas non-significant are in black. The Regulation of the actin cytoskeleton signalling pathway (p<0.0026) is shown in yellow. The size of the circle is proportional to the number of these genes in this pathway. The Regulation of the actin cytoskeleton signalling pathway (p<0.0026) was confirmed, where the proteins with different regulation were identified. Here, the RHOA, DIAPH1, RAC1, CYFP1, GNA13, IQGAP1 and ITGB1 were up-regulated, and GSN was down-regulated (Fig. 4).

### 3.5 MCF-7/WT and MCF-7/DOX-1 cells post-translational modifications

**Figure 5:**
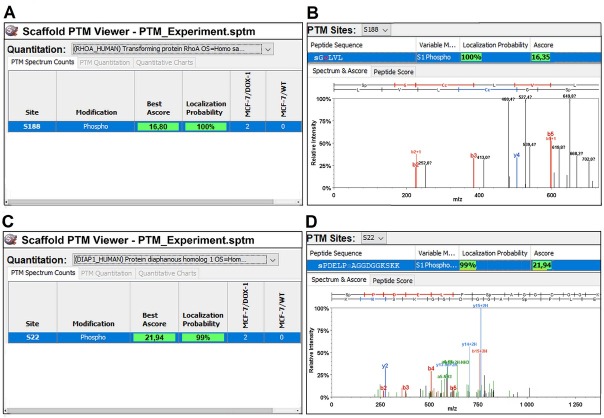
Quantification of post-translational modifications (PTMs) of RHOA and mDia1 in MCF-7/WT and MCF-7/DOX-1 cells. (A, C) Modified serine residues of RHOA, mDia1 and number of detected peptides with corresponding modification(s) observed in MCF-7/WT and MCF-7/DOX-1. (B, D) Sequence and mass spectrum of the peptide with phosphorylated residues (Ser-188 and Ser-22) located in RHOA and mDia1 proteins.

Evaluation of post-translational modifications using Scaffold PTM (Trial Version 3.3.0) revealed no RHOA and mDia1 phosphorylated serine peptides in MCF-7/WT cells (Fig. 5A. C). In contrast, two phosphorylated serine RHOA and mDia1 peptide (Ser-188 and Ser-22) (Ascore values of 16.35 and 21.94) were observed (at α < 0.01) in MCF-7/DOX-1 cells (Fig. 5B, D).

### 3.6 Structural modelling of phosphorylated serine 188 of RHOA-RhoGDIα complex

**Figure 6:**
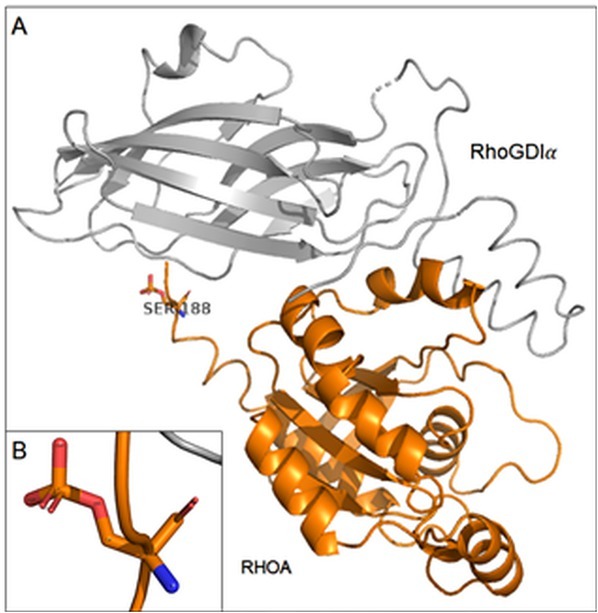
(A) Crystal structure of phosphorylated RHOA-RhoGDIα complex using PyTMs plugin in PyMOL software [PDB: 1CC0] (B) A detailed structure of phosphorylated serine 188.

It was observed that by the effect of the high dose of DOX (1μM) during 24 h period in MCF-7/DOX-1 cells, RHOA (serine 188) protein was phosphorylated which increases its interactions between RHOA and RhoGDI. When the RhoA is in an active state, the flexible C-terminal region provides a binding surface for specific downstream effector (mDia1) opposite in inactive state for RhoGDI [16]. The overall structure of the RHOA-RhoGDI complex in cartoon representation is shown in Fig. 6.

## 4 Discussion

At a concentration of 1 μM, DOX inhibits topoisomerase II [17], inducing its cytotoxicity [4, 18]. Kurbacher et al. detected a significant reduction of the viability of MCF-7 cells after high DOX concentration (1 μM and higher) [19]. Also, Xavier et al. determined, that the viability of the MCF-7 cells of treatment with DOX (1 μM) after 24 h was decreased (approximately 33 %) [20]. In our study, the cell viability was determined by the xCelligence (RTCA) system. Here, the normalised cell index (NCI = 2.03, time: 48h and 7min) of the MCF-7/WT cells (red line), was reduced by around 30 % by DOX in the MCF-7/DOX-1 cells (brown line; NCI = 1.38, time: 48h and 7min) after 24 h treatment (Fig. 1). Further, the cell viability was measured by flow cytometry. The obtained results also indicated a 30 % decrease in viability, of the MCF-7/DOX-1 cell (Fig. 2), which is in accordance with RTCA and the above outcomes.

**Figure 7:**
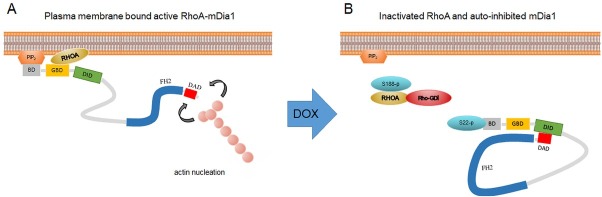
(A) Plasma membrane bound active RHOA-mDia1 in MCF-7/WT cells (B) Inactivated RHOA and auto-inhibited mDia1 in MCF-7/DOX-1 cells.

Doxorubicin is an anti-neoplastic agent known to induce actin cytoskeleton remodelling, i.e. stress fibre disruption [21]. The actin cytoskeleton plays an essential role in many cell processes, involving morphologic changes and cell apoptosis [22, 23]. In this paper, the regulation of the actin cytoskeleton through the signalling pathway was confirmed (p<0.0026), where RHOA and its downstream DIAPH1 proteins were up-regulated. These proteins participate in the regulation of actin cytoskeleton because their activation facilitates the polymerization of actin [24] which leads to the formation of filamentous actin (F actin) bundles called stress fibres [25, 26]. On the other hand, dysregulation of actin cytoskeleton, associated with the stress fibre disruption, that significantly contribute to apoptosis. It is a multi-molecular process that may be mediated by the inactivation of RHOA and auto-inhibition of mDia1. These claims were showed on the model system as depicted in (Fig. 7).

It is known, that mDia1 structure contains N-terminal basic region (BD), which interacts with the negatively charged phospholipids PIP2 during its activation in the plasma membrane [27]. Also, the second region of GTPase-binding domain (GBD) interacts with a membrane-bound RHOA [28, 29] (Fig. 7A). On the other hand, phosphorylation of RHOA at Ser-188 increases interactions of RHOA with RhoGDI and its inhibition to the mDia1 protein [12, 30] during DOX treatment. Further, by the phosphorylation of mDia1 at Ser-22 occurs to its autoinhibition, thus, mDia1 may adopt an intramolecular autoinhibitory conformation in which the N-terminal mDia1-inhibitory domain (DID) binds to the C-terminal mDia1 autoregulatory domain (DAD) as depicted in Fig. 7B [31, 32]. As a result of actin depolymerisation, stress fibre disruption and cell apoptosis come into existence.

## 5 Conclusions

The RTCA and flow cytometry results revealed DOX induced apoptosis of the MCF-7 cells. Also, phosphorylation of both the RHOA (Ser-188) and mDia1 (Ser-22) which are important to initialise the actin cytoskeletal remodelling (i.e., collapse actin stress fibre) were confirmed. Thus, our results pointed to an assumed role of the phosphorylated RhoA and mDia1 proteins in DOX-elicited dysregulation of actin cytoskeleton and cells death of the MCF-7/DOX-1 cells.
